# Noise constrains heterospecific eavesdropping more than conspecific reception of alarm calls

**DOI:** 10.1098/rsbl.2023.0410

**Published:** 2024-01-17

**Authors:** You Zhou, Andrew N. Radford, Robert D. Magrath

**Affiliations:** ^1^ Division of Ecology & Evolution, Research School of Biology, Australian National University, Canberra, Australian Capital Territory, Australia; ^2^ School of Biological Sciences, University of Bristol, Bristol, UK

**Keywords:** ambient noise, acoustic communication, heterospecific eavesdropping, alarm call, anti-predator behaviour, birds

## Abstract

Many vertebrates eavesdrop on alarm calls of other species, as well as responding to their own species' calls, but eavesdropping on heterospecific alarm calls might be harder than conspecific reception when environmental conditions make perception or recognition of calls difficult. This could occur because individuals lack hearing specializations for heterospecific calls, have less familiarity with them, or require more details of call structure to identify calls they have learned to recognize. We used a field playback experiment to provide a direct test of whether noise, as an environmental perceptual challenge, reduces response to heterospecific compared to conspecific alarm calls. We broadcast superb fairy-wren (*Malurus cyaneus*) and white-browed scrubwren (*Sericornis frontalis*) flee alarm calls to each species with or without simultaneous broadcast of ambient noise. Using two species allows isolation of the challenge of heterospecific eavesdropping independently of any effect of call structure on acoustic masking. As predicted, birds were less likely to flee to heterospecific than conspecific alarm calls during noise. We conclude that eavesdropping was harder in noise, which means that noise could disrupt information on danger in natural eavesdropping webs and so compromise survival. This is particularly significant in a world with increasing anthropogenic noise.

## 1. Introduction

Alarm calls warning of predators are important for many mammals and birds, with individuals often responding not only to conspecific alarm calls but also eavesdropping on those of other species [1,2]. Listeners respond with anti-predator behaviour appropriate to the type of alarm call, which likely enhances survival [3,4]. Heterospecific eavesdropping is taxonomically widespread, providing extra information on predators [5,6]. However, eavesdropping on other species’ calls has limitations, such as whether calls are familiar or whether they indicate a predator relevant to the listening species [7], and there can be reduced response even to familiar and relevant heterospecific alarm calls compared to conspecific equivalents [8,9]. Here we consider if environmental noise imposes greater limitations on response to alarm calls of other species compared to one's own.

Heterospecific eavesdropping may be harder than conspecific reception for several, not mutually exclusive, reasons. First, senders target signals to specific receivers, such as by orienting towards or approaching them, but eavesdroppers will not benefit from these adaptations [10,11]. For eavesdroppers, alarm calls therefore come from unpredictable distances and directions, requiring extra attention to detect calls [12]. Second, different species have different hearing abilities, and individuals can have species-specific perceptual specializations for their own vocalizations yet be poor at detecting other species' calls [13]. For example, great tits (*Parus major*) are more sensitive to higher frequencies than sparrowhawks (*Accipiter nisus*), making eavesdropping on great tit alarm calls difficult for sparrowhawks [14]. Such scenarios may also apply to species that eavesdrop on others for information about shared predators. Third, eavesdroppers can be less familiar with heterospecific compared to conspecific calls, potentially making it harder to recognize heterospecific calls that vary among individuals or are challenging to perceive [12]. For instance, European starlings (*Sturnus vulgaris*) were better at detecting familiar than unfamiliar songs during background noise [15]. Finally, individuals can respond to key features of conspecific alarm calls, while eavesdropping appears to require recognizing the overall structure of calls [16,17]. For example, superb fairy-wrens (*Malurus cyaneus*) use peak frequency to recognize conspecific aerial alarm calls, and so they also flee to unfamiliar aerial alarm calls if the peak frequency is similar to their own calls (9 kHz) [17]. However, while they can learn to recognize and flee to New Holland honeyeater (*Phylidonyris novaehollandiae*) alarm calls, which have different frequencies, they failed to respond if the calls were reversed but retained their peak frequency [18]. This suggests that eavesdropping requires more details of the signal, which may be obscured in poor conditions for listening.

Environmental noise provides a general constraint on acoustic communication [19,20], and so may exacerbate the challenge of heterospecific eavesdropping. Environmental noise is unavoidable, and worsening anthropogenic noise can threaten animal fitness, including by compromising reception of calls [19–21]. In natural environments, the effective range of a signal is often determined by noise and not hearing sensitivity [21,22]. Noise can reduce response by wild individuals to conspecific alarm calls, including by superb fairy-wrens and great tits [23,24], and to heterospecific alarm calls, as seen in dwarf mongooses (*Helogale parvula*) and Northern cardinals (*Cardinalis cardinalis*) [25,26]. However, we are aware of no field study testing whether noise affects heterospecific eavesdropping more severely than reception of conspecific calls. This is an important issue because noise may compromise the information web and so reduce the survival of individuals within communities.

We investigated whether noise constrains heterospecific eavesdropping on alarm calls more than conspecific reception of alarm calls. Our study species, superb fairy-wrens and white-browed scrubwrens (*Sericornis frontalis*), eavesdrop on each other's alarm calls [27]. Previous work in quiet conditions found that each species almost always fled to alarms from both species when broadcast at mean amplitudes, but were slightly less likely to respond to heterospecific alarms when amplitude was reduced [8,28]. This result is consistent with our prediction that heterospecific eavesdropping would become more difficult than conspecific alarm reception during noise.

## 2. Material and methods

**Figure 1.**
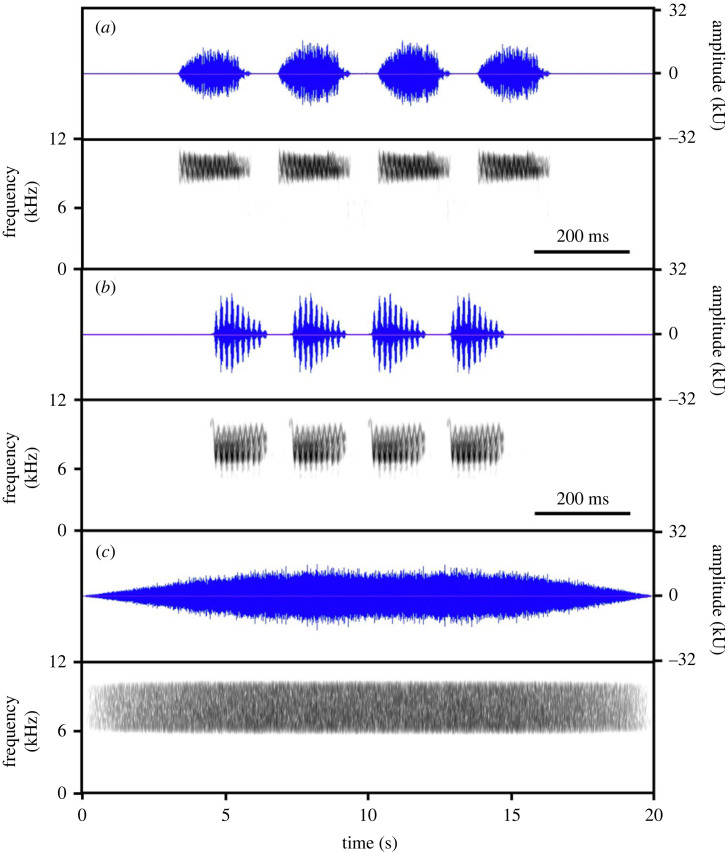
Examples of playback sounds: (*a*) fairy-wren alarm call; (*b*) scrubwren alarm call; and (*c*) filtered ambient noise; waveform is shown above, spectrogram below. Note the different timescale for alarm calls. The amplitude is on a linear scale, and expressed as the uncalibrated digital amplitude, given 16-bit wave files, where the absolute maximum is 32 768. Spectrograms were prepared in Raven Pro 1.5 (Blackman window type, 5.8 ms window size and 95% overlap).

We studied fairy-wrens and scrubwrens in the Australian National Botanic Gardens (−35.279°S, 149.109°E), Canberra. All fairy-wrens and most scrubwrens were colour-banded for long-term studies [29,30]. These small passerines have overlapping territories, and can join mixed-species flocks together [28,31,32]. They are vulnerable to the same predators, including locally common collared sparrowhawks (*Accipiter cirrhocephalus*) and pied currawongs (*Strepera graculina*) [18]. To warn of flying predators, both species give aerial alarm calls composed of repeated elements, each about 100 ms, with fairy-wren elements of higher peak frequency than those of scrubwrens (mean ± s.d.: 9.1 ± 0.4 versus 7.1 ± 0.4 kHz), and scrubwren elements having two bands at different frequencies (figure 1) [28]. For both species, calls with four elements prompt immediate flight to cover [28,33]. We conducted playbacks on adults and young at least one month old, so all were familiar with alarm calls from both species [30].

Our playback experiment investigated the effect of noise on heterospecific eavesdropping and conspecific reception, by broadcasting alarm calls of fairy-wrens and scrubwrens to both species either with or without broadcast of noise. Playback treatments were matched by location to control for any local variation in sound transmission and background noise. We tested fairy-wrens and scrubwrens at 16 locations within the study site, with each species at each location receiving all five playback treatments: noise alone (a control) and four alarm-call treatments, defined by the presence of noise (included or not) and caller species (fairy-wren or scrubwren). Fairy-wrens can move across locations but we ensured no individual received the same treatment more than once. We used this cross-species design to control for any characteristics that could make alarm calls of one species more vulnerable to acoustic masking. For example, if fairy-wrens fled less to scrubwren alarms than their own in noise, it could simply be that scrubwren alarms are more vulnerable to masking. But if this was so, scrubwrens would also flee less to their own alarms during noise. By contrast, we predict that noise will make each species less responsive to heterospecific than conspecific alarms.

Playback sounds were prepared using the same methods as in Zhou *et al*. [23]. In brief (details in electronic supplementary material), we made 16 playbacks of four-element alarm calls of each species, each from a different individual. Calls were broadcast at 52 dB SPL re 20 µPa (average amplitude of individual elements) at 10 m, which is about the mean natural amplitude for each species [28]. We combined these alarm calls with 16 ambient-noise sound files, each from separate recordings from the field site and filtered to 6–10 kHz, which covers the peak frequencies of alarm calls from both species (figure 1). Noise lasted for 20 s, fading in for 7 s and out for 5 s to avoid abrupt changes. Noise was calibrated to 52 dB SPL at 10 m, because at this level birds slightly reduced their response to conspecific alarm calls [23]. Furthermore, this level is well above environmental noise on the dry, still, winter days when playbacks were carried out (mean ± SD measured immediately after each playback: 38.3 ± 2.9 dB SPL; range 30.4–46.3 dB SPL; *n* = 159, one measurement missing; details below), but below maximum natural levels recorded at the study site of 54–64 dB SPL on windy days. For treatments including noise, the alarm and noise audio files were mixed into one file, with alarm calls starting during the stable period of noise.

Playbacks were carried out from June to August 2017. Playback methods followed our previous work on these species [18,23,28,34], in which we use a mobile playback system consisting of a Roland Edirol R-05 HR digital recorder, a custom amplifier and a Peerless 810921 tweeter loudspeaker (frequency response 2–11 kHz). Sixteen locations were selected in the Botanic Gardens for each species, featuring open areas where birds frequently foraged. For each playback, a focal individual was followed by the observer (Y.Z.) from about 10 m, and had at least 5 min of undisturbed foraging before playback. If there were natural alarm calls, the clock was reset for another 5 min. Playbacks were carried out when there were no heterospecifics nearby, and the focal bird was foraging on the ground about 10 m from the observer and 0.5–10 m from cover. These are social birds, and conspecifics were present during 76 out of 160 playbacks, but the focal bird was always the closest individual. Furthermore, we found no effect of conspecific presence or distance to cover on the likelihood of focal birds fleeing to alarm calls (electronic supplementary material). We ensured there was no obstruction between the loudspeaker and the focal bird that could affect sound transmission or obscure our view of the bird's response. Playbacks were carried out in relatively quiet periods when there was no prominent background sound, such as from aircraft or nearby vehicles. Following playback, the observer recorded whether the focal bird fled to cover, the almost invariant response by both species to playback of multi-element aerial alarm calls broadcast at mean amplitude under quiet conditions [18,23]. Fleeing was defined as stopping foraging, and flying immediately and directly to cover in nearby vegetation. Then, after recording the bird's response, the natural background sound was audio-recorded for 30 s to obtain an estimate of background noise level, using the same equipment used to record the noise for playbacks. The background noise level was measured using average power function in Raven 1.5 Pro and calibrated against a tone of known amplitude.

We used McNemar tests to compare the responses to heterospecific and conspecific alarm calls with and without noise. McNemar tests compare the difference in probabilities of paired dichotomous variables, in our case flee versus not flee, matched by location and receiver species [35,36]. We analysed the playbacks to fairy-wrens and scrubwrens together with 32 location-species pairs, because any effect of receiver species was controlled in the crossover experimental design. The effect of location and treatment order were also controlled as the experiment used a complete block design. McNemar tests are suitable when probabilities are on different parts of the 0–1 spectrum, as in our data, which is problematic for binomial mixed models using log odds ratios [37,38]. Our experiment was motivated by our previous results and was designed to test the directional hypothesis that heterospecific eavesdropping is harder than conspecific reception, especially during noise. Nonetheless, we follow a common but not universal convention of using two-tailed tests [36,39,40]. First, we examined responses within noisy and quiet conditions separately, predicting a reduced response to heterospecific compared to conspecific alarms, particularly during noise. Second, we directly compared responses in noisy versus quiet conditions, predicting that the reduced response to heterospecific alarms would be greater in noise compared to quiet conditions.

## 3. Results

**Figure 2.**
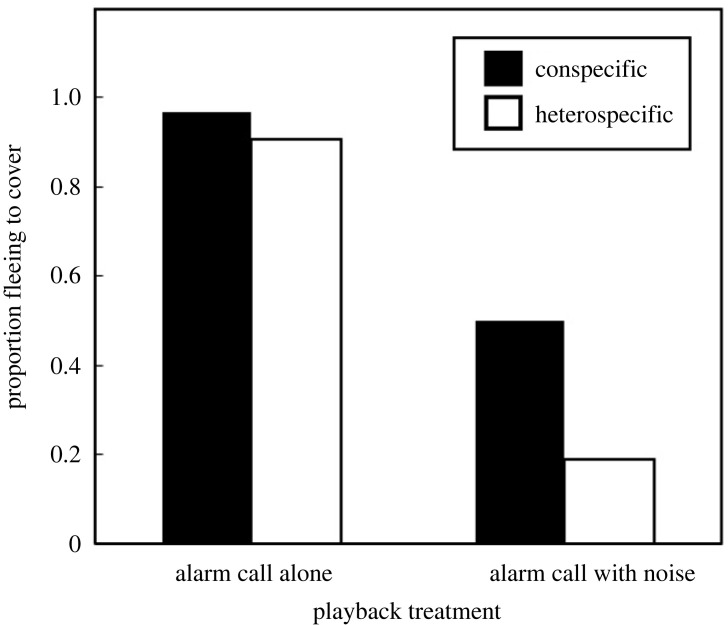
Proportion of fairy-wrens and scrubwrens that fled to cover to playback of conspecific and heterospecific alarm calls alone and with added noise (*N* = 32 birds, with 16 of each species, for each treatment). Statistical analyses are in the Results.

As predicted, noise disproportionately affected response of birds to heterospecific compared to conspecific alarm calls (figure 2). Noise itself appeared to have minimal impact on birds; for noise-alone playbacks, no bird fled during the stable period of noise (when the alarm call potentially occurred in the alarm-with-noise treatments), and only two birds fled to cover at the onset of noise (electronic supplementary material, table S1). Without noise, almost all birds fled to cover after alarm calls and there was no significant difference between fleeing to conspecific and heterospecific alarm calls (McNemar test, exact two-tailed: three fled to conspecific but not heterospecific alarm calls versus one the opposite, *p* = 0.625; electronic supplementary material, tables S1, S2). However, birds fled less to heterospecific than conspecific alarm calls when noise was broadcast simultaneously (11 fled to conspecific but not heterospecific alarm calls, with only one the opposite, *p* = 0.006; electronic supplementary material, tables S1, S2). Furthermore, there was a strong trend that the difference between fleeing to heterospecific and conspecific alarm calls was larger when noise was played than not played (10 birds showed a greater reduction in response in noisy conditions compared to quiet conditions versus three the opposite, *p* = 0.092; electronic supplementary material, tables S1, S2).

## 4. Discussion

Noise compromised response to heterospecific alarm calls more than to conspecific alarms. When noise was not broadcast, almost all birds fled to alarm calls from both species, and the proportions fleeing to conspecific and heterospecific alarms were similar. However, during noise individuals were more likely to flee to conspecific alarms than those from the other species. Furthermore, the difference of fleeing probabilities between conspecific and heterospecific alarms tended to be greater with noise than without noise, and the strong trend was consistent with our directional hypothesis. To our knowledge, this is the first experimental test of the effect of noise on heterospecific eavesdropping compared to conspecific alarm-call reception.

Our cross-species experiment allowed us to distinguish the constraint of heterospecific eavesdropping from the possibility that differences in call structure alone affected masking and response. Our results show that, as predicted, heterospecific eavesdropping was more difficult than conspecific communication in noisy conditions. By contrast, a previous laboratory study on budgerigars (*Melopsittacus undulatus*) and zebra finches (*Taeniopygia guttata*) found that contact call structure affected hearing thresholds in noise, not whether the call was from a heterospecific or conspecific [41]. In that study, call structures were very different between species, and both species were better at detecting calls of budgerigars than zebra finches during noise playback, potentially because of differences in tonality and peak amplitude [42]. Our experiment was carried out in the wild and the calls were similar in structure, and so likely to be similarly vulnerable to masking. This may explain why we detected the pattern of reduced response to heterospecific alarm calls, rather than an effect of call structure.

Species-specific hearing adaptions, the more challenging recognition of learned calls or selective attention to conspecific calls most likely explain our results. First, superb fairy-wrens have higher frequency aerial alarm calls (9 kHz peak frequency) than those of white-browed scrubwrens (7 kHz). Each species' perceptual abilities may be best at detecting the prominent frequency of conspecific alarms during noise. Second, fairy-wrens and scrubwrens learn to recognize the other species’ aerial alarm calls [18,30,43] and recognizing learned calls is likely to be harder in noise. Learned recognition of heterospecific calls appears to require recognition of acoustic details, such as duration, interval and frequency modulation, rather than key features like peak frequency [16,17]. Consequently, noise may mask acoustic details that are important for heterospecific eavesdropping but not for conspecific reception that may rely on simple features resistant to degradation [8,17]. Third, animals typically attend to the most relevant information amongst simultaneous sources [44]. Selective attention might therefore also cause a greater response to conspecific than heterospecific calls during noise.

In conclusion, our study shows that eavesdropping was harder than conspecific reception during the challenge of environmental noise. If this finding applies broadly among species, then noise will have a disproportionate effect on heterospecific eavesdropping compared to conspecific reception, and thereby compromise the ‘information web’ in natural communities [1,45,46]. Our experiment was designed to mimic a natural situation, but could potentially overestimate or underestimate the effect of noise in natural communities. On the one hand, we used a single loudspeaker so that the alarm call and noise came from the same direction, but in natural communities the signal and noise could come from different directions, which could reduce the strength of masking (‘spatial release of masking’; [47]). On the other hand, we directed our playbacks towards nearby focal individuals, whereas in natural circumstances only conspecific alarm calls are likely to be directed to a specific listener. By contrast, heterospecific calls come from unpredictable distances and directions, with calls often attenuated and degraded, so potentially exacerbating the effects of noise [1]. Consequently, our experiment may have underestimated the relative difficulty of heterospecific eavesdropping during noise. Given the conservation challenges of increasing anthropogenic noise [47–50], understanding the effects on different species in the community is ecologically important for assessing the broad impact of noise and predicting potential outcomes of noise exposure.

## Data Availability

The original data of this paper are presented in the electronic supplementary material, table S1 and electronic supplementary material, table S3 [51].
